# Risk factors associated with nasopharyngeal carriage and density of *Streptococcus pneumoniae*, *Haemophilus influenzae*, *Moraxella catarrhalis*, and *Staphylococcus aureus* in young children living in Indonesia

**DOI:** 10.1186/s41479-018-0058-1

**Published:** 2018-12-25

**Authors:** Eddy Fadlyana, Eileen M. Dunne, Kusnandi Rusmil, Rodman Tarigan, Sunaryati Sudigdoadi, Chrysanti Murad, Emma Watts, Cattram Nguyen, Catherine Satzke, Nurhandini Eka Dewi, Sang Ayu Kompiyang Indriyani, Finny Fitry Yani, Kim Mulholland, Cissy Kartasasmita

**Affiliations:** 1 Department of Child Health, Universitas Padjadjaran/Hasan Sadikin General Hospital, Bandung, West Java Indonesia; 2 Pneumococcal Research, Murdoch Children’s Research Institute, Parkville, VIC Australia; 3 Department of Paediatrics, The University of Melbourne, Parkville, VIC Australia; 4 Department of Biomedical Sciences, Division of Microbiology, Universitas Padjadjaran, Bandung, West Java Indonesia; 5 Department of Microbiology and Immunology, The University of Melbourne at the Peter Doherty Institute for Infection and Immunity, Parkville, VIC Australia; 6 District Health Office of Central Lombok, Praya, West Nusa Tenggara Indonesia; 7 West Nusa Tenggara Province General Hospital, Mataram, West Nusa Tenggara Indonesia; 8 Department of Child Health, Universitas Andalas, Padang, West Sumatra Indonesia; 9 London School of Hygiene and Tropical Medicine, London, UK

**Keywords:** Nasopharynx, Risk factors, Stunting, Carriage, Density, *Streptococcus pneumoniae*, Pneumococcus, *Haemophilus influenzae*, *Moraxella catarrhalis*, and *Staphylococcus aureus*

## Abstract

**Background:**

Potentially pathogenic bacteria *Streptococcus pneumoniae*, *Haemophilus influenzae*, *Moraxella catarrhalis*, and *Staphylococcus aureus* are commonly carried in the nasopharynx of young children. Host and environmental factors have been linked with pathogen carriage, and in many studies rural children have higher carriage rates than their urban counterparts. There are few published data on what factors contribute to increased pathogen density. The objectives of this study were to identify risk factors for nasopharyngeal carriage and density of *S. pneumoniae*, *H. influenzae*, *M. catarrhalis*, and *S. aureus* in young children in Indonesia.

**Methods:**

Risk factor analysis was done using data on bacterial carriage and participant characteristics from a cross-sectional study that enrolled 302 children aged 12–24 months living in urban or semi-rural areas of Indonesia. Associations between host factors and odds of pathogen carriage were explored using logistic regression. Characteristics identified to be independent predictors of carriage by univariable analysis, as well as those that differed between urban and semi-rural participants, were included in multivariable models. Risk factors for increased pathogen density were identified using linear regression analysis.

**Results:**

No differences in carriage prevalence between urban and semi-rural children were observed. Multiple children under the age of 5 years in the household (< 5y) and upper respiratory tract infection (URTI) symptoms were associated with *S. pneumoniae* carriage, with adjusted odds ratios (aOR) of 2.17 (95% CI 1.13, 4.12) and 2.28 (95% CI 1.15, 4.50), respectively. There was some evidence that URTI symptoms (aOR 1.94 [95% CI 1.00, 3.75]) were associated with carriage of *M. catarrhalis*. Children with URTI symptoms (*p* = 0.002), and low parental income (*p* = 0.011) had higher *S. pneumoniae* density, whereas older age was associated with lower *S. pneumoniae* density (*p* = 0.009). URTI symptoms were also associated with higher *M. catarrahlis* density (*p* = 0.035). Low maternal education (*p* = 0.039) and multiple children < 5y (*p* = 0.021) were positively associated with *H. influenzae* density, and semi-rural residence was associated with higher *S. aureus* density (*p* < 0.001).

**Conclusions:**

This study provides a detailed assessment of risk factors associated with carriage of clinically-relevant bacteria in Indonesian children, and new data on host factors associated with pathogen density.

**Electronic supplementary material:**

The online version of this article (10.1186/s41479-018-0058-1) contains supplementary material, which is available to authorized users.

## Background

The nasopharynx of young children is commonly colonized by potentially pathogenic bacteria including *Streptococcus pneumoniae* (the pneumococcus), *Haemophilus influenzae*, *Moraxella catarrhalis*, and *Staphylococcus aureus*. *S. pneumoniae*, *H. influenzae*, and *S. aureus* are major causes of pneumonia, the second most common killer (behind preterm birth complications) of children under the age of five years worldwide [1, 2]. *S. pneumoniae*, *H. influenzae*, and *M. catarrhalis* are the leading etiologic agents associated with otitis media [3]. Carriage of these species is generally asymptomatic, but particularly for *S. pneumoniae*, carriage is considered a prerequisite for disease as well as the source of transmission [4]. *S. pneumoniae* carriage prevalence varies from 19 to 86% depending on factors such as age, geographic location, and HIV status [4]. A meta-analysis reported a pooled prevalence estimate of 47.8% for pneumococcal carriage in children under five in lower- middle income countries and 64.8% in low income countries [5]. PCR detection of *H. influenzae* carriage in healthy children from low and lower-middle income countries ranges from 31 to 70% [5]. Reported carriage rates of *M. catarrhalis* in children under two years of age range from 25 to 76% [6, 7]. *S. aureus* carriage is highest in early infancy and older children, and typically low (prevalence < 10%) in children aged 12–24 months [6–8].

Several host and environmental factors have been associated with increased risk of *S. pneumoniae* carriage in children, including day care attendance, having siblings or living with other young children, symptoms of respiratory infection, and low socio-economic status [9–13]. Additionally, children in rural areas have been found to have higher *S. pneumoniae* carriage rates than children in urban settings [14, 15]. Symptoms of respiratory infection and day-care attendance have been identified as risk factors for *H. influenzae* carriage in children, and low socio-economic status and parents who smoke for *M. catarrhalis* [11, 13, 16]. In contrast, risk factors typically associated with *S. pneumoniae* carriage, such as day-care attendance, having younger siblings, or living in a rural area, are associated with reduced risk of *S. aureus* carriage [5, 9]. In studies including HIV-positive children, HIV infection was a risk factor for *S. aureus* carriage, whereas associations between HIV status and *S. pneumoniae* carriage have varied [17–19]. In HIV-infected children, respiratory infection has been identified as a risk factor for *S. pneumoniae* carriage, coryza and school attendance were linked to higher *S. aureus* carriage, and *M. catarrhalis* carriage was more common in children from urban settings compared to rural, as well as those whose mothers had higher levels of education [20, 21].

In addition to the presence of a potential pathogen in the nasopharynx, the quantitative bacterial load (density) has been increasingly recognized as clinically important. High *S. pneumoniae* density in the nasopharynx has been associated with respiratory infection and pneumonia in children, and linked to transmission in animal studies [22, 23]. Nasopharyngeal density of *S. pneumoniae*, *H. influenzae*, and *M. catarrhalis* was found to be higher in children with otitis media compared to healthy controls [24]. There are few published data on risk factors associated with increased bacterial density in the nasopharynx, however co-infection with respiratory viruses is associated with increased density of *S. pneumoniae* and *H. influenzae* [24, 25].

Indonesia is a lower-middle income country with an estimated population of over 260 million, the world’s fourth highest (www.worldbank.org). Pneumonia is the most common cause of death in the post-neonatal period in Indonesian children, and in 2013 was estimated to kill 22,000 children [26]. Indonesia is a diverse country composed of over 17,000 islands. Childhood mortality varies significantly among island regions and between urban and rural areas [27]. Vaccines against *H. influenzae* type B (Hib) were introduced in Indonesia in 2013. Pneumococcal conjugate vaccines are not yet part of the national immunization program, although a government-led demonstration program of PCV13 commenced in late 2017 in West Nusa Tenggara. Previously, we conducted a cross-sectional carriage study in children 12–24 months of age in three Indonesian regions and reported a carriage prevalence of 49.5% for *S. pneumoniae*, 27.5% for *H. influenzae*, 42.7% for *M. catarrhalis*, and 7.3% for *S. aureus* [28]. No Hib carriage was detected due to vaccine use. The *S. pneumoniae* carriage rate was similar to previous reports from Central Java and Lombok, which ranged from 43 to 48% [29–31]. In Semarang, Central Java, *S. pneumoniae* carriage rates varied by district, with higher rates found in suburban areas [29]. Here, we examine risk factors for pathogen carriage and density in children from semi-rural and urban areas using data from this cross-sectional study. We hypothesized that children from semi-rural areas would have higher odds of pathogen carriage.

## Methods

### Study design and participants

Details on the study participants and laboratory methods were previously published [28]. In brief, 302 healthy children aged 12–24 months were enrolled at six health centers located in three different regions of Indonesia: Bandung, West Java; Lombok, West Nusa Tenggara, and Padang, West Sumatra. Half of the children were from urban communities and the other half from semi-rural areas. Recruitment was conducted by health center staff, who invited age-eligible children and their parents to participate. Inclusion criteria were age 12–24 months and residence within the health center jurisdiction. Exclusion criteria were moderate or severe acute illness, temperature ≥ 38 °C, antibiotic use within the previous 14 days, or previous receipt of pneumococcal conjugate vaccine.

For each participant, a medical examination was conducted by a pediatrician, and data on demographic characteristics, living conditions, and significant medical history were recorded on a case report form. Anthropometric measurements (Z scores) were calculated in Stata version 14.2 (StataCorp, College Station, TX, USA) using the Stata macro available online from the World Health Organization (WHO; http://www.who.int/childgrowth/software/en/, accessed 7 March 2018), with stunting defined as length-for-age z-score below − 2 standard deviations of the median length-for-age of the WHO Child Growth Standards [32]. A nasopharyngeal swab was collected according to WHO recommendations and placed immediately into 1 mL skim milk tryptone glucose glycerol media (STGG) media [33]. Swabs were kept in a cool box and transported to a local laboratory within 6 h of collection for aliquotting and storage at − 70 °C until use. Swabs from Lombok and Padang regions were shipped to the central laboratory in Bandung on dry ice, then stored at − 70 °C.

### Laboratory analyses

DNA was extracted from 200 μl of STGG sample using a QIAcube HT instrument (Qiagen) following enzymatic lysis. Bacteria were pelleted by centrifugation at 5500 x g for 8 min and lysis was conducted by incubation in a 20 mM Tris-HCl, 2 mM sodium EDTA buffer containing 20 mg/ml lysozyme, 1% (*v*/v) Triton X-100, 0.075 mg/ml mutanolysin and 2 mg/ml RNase A for 60 min at 37 °C, followed by the addition of proteinase K and Buffer AL from the QIAamp 96 DNA QIAcube HT Kit (Qiagen) and 30 min incubation at 56 °C. Lysates were transferred onto the QIAcube HT instrument (Qiagen) and DNA extraction performed according to the manufacturer’s instructions.

*S. pneumoniae* was detected and quantified using a real-time quantitative PCR (qPCR) assay targeting the *lytA* gene with confirmation by culture on sheep blood agar containing 5 μg/ml gentamicin [34]. A commercial kit (FTD Bacterial Pneumonia CAP qPCR kit; Fast-Track Diagnostics) was used for detection and quantification of *H. influenzae*, *M. catarrhalis*, and *S. aureus*. Full details are available in the Additional file 1.

### Statistical analysis

Data from case report forms was entered into a database (dBASE software, dBase LLC, Binghamton, NY, USA) and laboratory data entered into Microsoft Excel 2013. Datasets were imported, merged, and cleaning conducted using Stata version 14.2 (StataCorp, College Station, TX, USA), and statistical analyses were conducted using Stata version 14.2. To compare data between urban and semi-rural participants, the chi-squared test was used for categorical data and the t-test for continuous data following assesment of normality. For examination of potential risk factors for carriage, univariable odds ratios and 95% confidence intervals (CIs) were calculated using logistic regression. The following variables (shown in Table 1) were assessed: region, sex, residence (urban or semi-rural), age, stunting (defined as length-for-age z-score below − 2), maternal education, income, having two or more children under five years old in the household, upper respiratory tract infection (URTI) symptoms (rhinorrhea, cough, and/or tonsillitis), exposure to cigarette smoke, and presence of a wood-fuelled stove in the home. Two categorical variables were reclassified into binary variables prior to analysis: maternal education (below high school and high school or above) and income (at or below regional minimum salary or above regional minimum salary). Regional minimum salary rates in 2016 were 1,800,725 Indonesian rupiah (IDR) in Padang, 2,626,940 IDR in Bandung, and 1,550,000 IDR in Lombok. Paternal education was not included in analysis due to co-linearity with maternal education. Carriage of other colonizing pathogens was also included in risk factor evaluation.

**Table 1:** Characteristics of study participants

Characteristics	All participants (total = 302) N (%)	Urban (*n* = 152) N (%)	Semi-rural (*n* = 150) N (%)	*P* value^1^
Region				
Bandung	100 (33.1)	50 (32.9)	50 (33.3)	0.997
Lombok	101 (33.4)	51 (33.6)	50 (33.3)	
Padang	101 (33.4)	51 (33.6)	50 (33.3)	
Sex				
Male	158 (52.3)	82 (53.9)	76 (50.7)	0.568
Female	144 (47.7)	70 (46.0)	74 (49.3)	
Age (months)				
Mean ± SD	18.9 ± 3.3	18.8 ± 3.3	18.9 ± 3.3	0.852
Min; Max	12.3; 24.9			
Weight (kg)				
Mean ± SD	9.5 ± 1.4	9.4 ± 1.3	9.5 ± 1.4	0.472
Min; Max	6.4; 15.0			
Length (cm)				
Mean ± SD	78.8 ± 4.2	79.0 ± 4.1	78.5 ± 4.4	0.287
Min; Max	67; 91			
Length-for-age Z score				
Mean ± SD	−1.20 ± 1.22	−1.11 ± 1.20	−1.30 ± 1.22	0.175
Min; Max	−4.33; 4.19	−3.81; 4.19	−4.33; 1.65	
Stunting^2^				
No	221 (73.2)	116 (76.3)	105 (70.0)	0.215
Yes	81 (26.8)	36 (23.7)	45 (30.0)	
Paternal education				
None	10 (3.3)	5 (3.3)	5 (3.3)	0.739
Elementary school	32 (10.6)	15 (9.9)	17 (11.3)	
Junior high school	61 (20.2)	28 (18.4)	33 (22.0)	
Senior high school	155 (51.3)	78 (51.3)	77 (51.3)	
University	44 (14.6)	26 (17.1)	18 (12.0)	
Maternal education				
None	11 (3.6)	7 (4.6)	4 (2.7)	0.470
Elementary school	30 (9.9)	13 (8.6)	17 (11.3)	
Junior high school	78 (25.8)	34 (22.4)	44 (29.3)	
Senior high school	147 (48.7)	79 (52.0)	68 (45.3)	
University	36 (11.9)	19 (12.5)	17 (11.3)	
Parental monthly income^3^				
Declined to answer	2 (0.7)	0 (0.0)	2 (1.3)	0.041
< 500,000 IDR	43 (14.2)	29 (19.1)	43 (14.2)	
500,000 IDR - Regional minimum salary	167 (55.3)	77 (50.7)	90 (60.0)	
> Regional minimum salary	90 (29.8)	46 (30.3)	44 (29.3)	
Number of children <5y in the household^4^				
1	237 (78.5)	114 (76.5)	123 (82.0)	0.378
2	48 (15.9)	27 (18.1)	21 (14.0)	
3	12 (4.0)	6 (4.0)	6 (4.0)	
4	2 (0.7)	2 (1.3)	0 (0.0)	
Exposure to indoor cigarette smoke				
No	163 (54.0)	98 (64.5)	65 (43.3)	0.0004
Yes	139 (46.0)	54 (35.5)	85 (56.7)	
Wood-fuelled stove in home				
No	268 (88.7)	146 (96.0)	122 (81.3)	0.0001
Yes	34 (11.3)	6 (4.0)	28 (18.7)	
URTI symptoms^5^				
No	240 (79.5)	127 (83.6)	113 (75.3)	0.077
Yes	62 (20.5)	25 (16.4)	37 (24.7)	

^1^Chi-squared test for categorical data; t-test for continuous data

^2^Stunting (chronic undernutrition) defined as length-for-age Z score below −2

^3^*IDR* Indonesian rupiah; Regional minimum salary rates (2016) were 1,800,725 IDR in Padang, 2,626,940 IDR in Bandung, and 1,550,000 IDR in Lombok

^4^Data missing from three urban participants (*n* = 299 total, *n* = 149 urban)

^5^Upper respiratory tract infection (URTI) symptoms include rhinorrhea, cough, and/or tonsillitis

Multivariable logistic regression models were created for each pathogen to estimate adjusted odds ratios and 95% CI. Multivariable models included the following variables: residence (selected a priori), variables that varied between urban and semi-rural participants (income, exposure to cigarette smoke, having a wood-fuelled stove), and any other variables with *p* < 0.1 by univariable analysis.

Bacterial density data were log_10_ transformed prior to analysis and reported as log_10_ genome equivalents/ml (log_10_ GE/ml). Linear regression was used to examine relationships between potential risk factors and bacterial density, with analyses restricted to positive carriers for each species. The same variables used for risk factors for carriage were queried for association with density. Results were reported as linear regression coefficients and 95% CI. Covariates for the multivariable linear regression models used to estimate adjusted coefficients included variables with *p* < 0.1 by univariable analysis as well as residence type, income, and having a wood-fuelled stove.

## Results

**Fig. 1:**
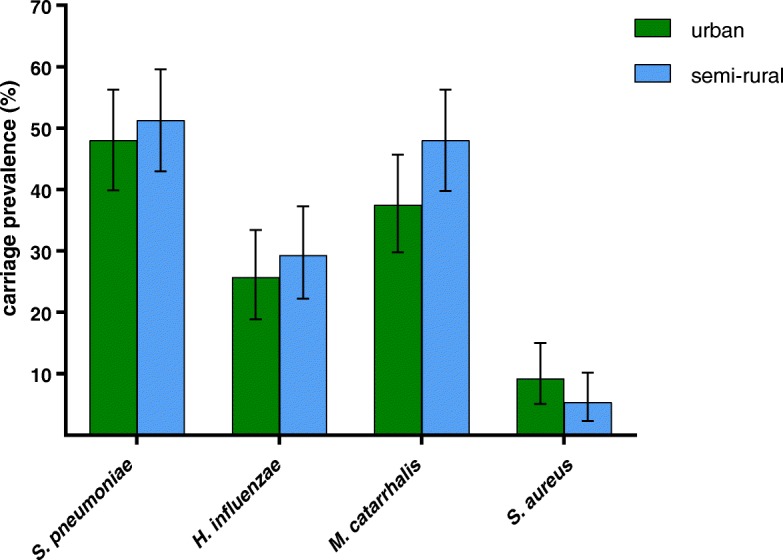
Nasopharyngeal carriage prevalence of potentially pathogenic bacteria in Indonesian children aged 12–24 months living in urban (green) or semi-rural (blue) areas. Error bars depict 95% confidence intervals

Three hundred and two children aged 12–24 months were included in this study, with 152 participants from urban areas and 150 from semi-rural areas. The characteristics of the study participants are shown in Table 1. Most characteristics were similar between the urban and semi-rural children, except that exposure to indoor cigarette smoke and having a wood-fuelled stove were higher for semi-rural children, and the income distribution differed between the two groups. The carriage prevalence for *S. pneumoniae*, *H. influenzae*, *M. catarrhalis*, and *S. aureus* for urban and semi-rural participants is shown in Fig. 1. There were no significant differences in carriage prevalence between urban and semi-rural children for *S. pneumoniae*, *H. influenzae*, or *S. aureus* (*p* > 0.10 for each). There was some evidence that carriage prevalence of *M. catarrhalis* was higher in semi-rural children (*p* = 0.065). 101 (33.4%) study participants carried one of the four bacterial species examined, and 125 (41.2%) carried multiple species. There was no difference in multiple species carriage prevalence between urban and semi-rural children (*p* = 0.496).

The relationships between participant characteristics and carriage of each species were examined using logistic regression analysis. For *S. pneumoniae*, univariable analysis found carriage differed between regions, with children in Bandung and Lombok having higher odds of pneumococcal carriage compared to children in Padang (Table 2). Low maternal education, stunting, the presence of URTI symptoms, and carriage of *M. catarrhalis* were significantly associated with increased odds of pneumococcal carriage. Following multivariable analysis region, living in a household with two or more children under the age of five, URTI symptoms, and *M. catarrhalis* carriage remained significant. For *M. catarrhalis*, carriage varied by region, with higher odds reported for children in Lombok compared to Padang (Table 2). Following multivariable analysis, URTI symptoms, *S. pneumoniae* carriage, and *H. influenzae* carriage were positively associated with *M. catarrhalis* carriage, whereas *S. aureus* carriage was negatively associated. Following adjustment, semi-rural residence was not associated with *M. catarrhalis* carriage (*p* = 0.205). There were no demographic factors that were significantly associated with carriage of *H. influenzae* or *S. aureus*, however carriage of *M. catarrhalis* was positively associated with *H. influenzae* and negatively associated with *S. aureus* (Additional file 1: Table S1). Potential risk factors for carriage of more than one species were also examined, and URTI symptoms had a strong positive association (aOR 3.2 [95%CI 1.69, 6.11]) by multivariable analysis (Additional file 1: Table S2).

**Table 2:** Univariable and multivariable analysis of risk factors for *S. pneumoniae* and *M. catarrhalis* carriage

Variable	*S. pneumoniae*					*M. catarrhalis*				
	carriers/total (%)	Unadjusted OR^a^ (95% CI)	*P* value	Adjusted OR^b^ (95% CI)	*P* value	carriers/total (%)	Unadjusted OR (95% CI)	*P* value	Adjusted OR^c^ (95% CI)	*P* value
Region										
Padang	35/101 (34.6)	reference	< 0.001	reference	0.040	32/101 (31.7)	reference	0.016	reference	0.046
Bandung	64/100 (64.0)	3.35 (1.88, 5.98)		2.46 (1.22, 4.95)		45/100 (45.0)	1.76 (0.99, 3.14)		1.06 (0.51, 2.16)	
Lombok	51/101 (50.5)	1.92 (1.09, 3.39)		1.49 (0.75, 2.94)		52/101 (51.5)	2.29 (1.29, 4.06)		2.15 (1.08, 4.27)	
Sex										
Female	76/144 (52.8)	reference	0.303			62/144 (43.1)	reference	0.909		
Male	74/158 (46.8)	0.79 (0.81, 1.24)				67/158 (42.4)	0.97 (0.62, 1.24)			
Residence										
Urban	73/152 (48.0)	reference	0.566	reference	0.982	57/152 (37.5)	reference	0.066	reference	0.430
Semi-rural	77/150 (51.3)	1.14 (0.73, 1.79)		1.01 (0.59, 1.72)		72/150 (48.0)	1.54 (0.97, 2.43)		1.24 (0.72, 2.14)	
Age (months)		1.02 (0.95, 1.09)	0.637				0.98 (0.91, 1.05)	0.582		
Stunting^d^										
No	101/221 (45.7)	reference	0.024	reference	0.884	82/221 (37.1)	reference	0.001	reference	0.083
Yes	49/81 (60.5)	1.82 (1.08, 3.05)		1.05 (0.56, 1.96)		47/81 (58.0)	2.34 (1.40, 3.94)		1.74 (0.93, 3.26)	
Maternal education										
Below high school	68/119 (57.1)	reference	0.037	reference	0.123	65/119 (54.6)	reference	0.001	reference	0.232
High school and above	82/183 (44.8)	0.61 (0.38, 0.97)		0.64 (0.36, 1.13)		64/183 (35.0)	0.45 (0.28, 0.72)		0.71 (0.40, 1.25)	
Parental monthly income^e^										
≤ Regional minimum salary	100/210 (47.6)	reference	0.279	reference	0.242	92/210 (43.8)	reference	0.430	reference	0.944
> Regional minimum salary	49/90 (54.4)	1.31 (0.80, 2.16)		1.55 (0.84, 2.83)		35/90 (38.9)	0.82 (0.49, 1.35)		0.98 (0.53, 1.81)	
Children <5y in the household										
1	112/237 (47.3)	reference	0.051	reference	0.019	100/237 (42.2)	reference	0.848		
2 or more	38/62 (61.3)	1.77 (1.00, 3.13)		2.17 (1.13, 4.12)		27/62 (43.6)	1.06 (0.60, 1.86)			
URTI symptoms^f^										
No	108/240 (45.0)	reference	0.002	reference	0.018	92/240 (38.3)	reference	0.003	reference	0.050
Yes	42/62 (67.7)	2.57 (1.42, 4.63)		2.28 (1.15, 4.50)		37/62 (59.7)	2.38 (1.35, 4.21)		1.94 (1.00, 3.75)	
Exposure to cigarette smoke										
No	87/163 (53.4)	reference	0.164	reference	0.242	69/163 (42.3)	reference	0.884	reference	0.375
Yes	63/139 (45.3)	0.72 (0.46, 1.14)		0.72 (0.42, 1.24)		60/139 (43.2)	1.04 (0.66, 1.64)		1.28 (0.74, 2.20)	
Wood-fuelled stove in home										
No	133/268 (49.6)	reference	0.967	reference	0.623	113/268 (42.2)	reference	0.587	reference	0.852
Yes	17/34 (50.0)	1.02 (0.50, 2.07)		1.23 (0.53, 2.86)		16/34 (47.1)	1.23 (0.60, 2.50)		0.92 (0.39, 2.18)	
*M. catarrhalis* carriage						*S. pneumoniae* carriage				
No	66/173 (38.2)	reference	< 0.001	reference	< 0.001	45/152 (29.6)	reference	< 0.001	reference	0.001
Yes	84/129 (65.1)	3.03 (1.88, 4.86)		2.64 (1.54, 4.50)		84/150 (56.0)	3.03 (1.88, 4.86)		2.52 (1.48, 4.28)	
*H. influenzae* carriage										
No	102/219 (46.6)	reference	0.082	reference	0.454	81/219 (37.0)	reference	0.001	reference	0.023
Yes	48/83 (57.8)	1.57 (0.94, 2.62)		1.24 (0.70, 2.21)		48/83 (57.8)	2.34 (1.40, 3.91)		1.94 (1.09, 3.43)	
*S. aureus* carriage										
No	142/280 (50.7)	reference	0.200			128/280 (45.7)	reference	0.005	reference	0.016
Yes	8/22 (36.4)	0.56 (0.23, 1.36)				1/22 (4.6)	0.06 (0.01, 0.43)		0.08 (0.01, 0.63)	

^a^*OR* odds ratio

^b^Adjusted for region, residence type, income, maternal education, 2 or more children <5y, upper respiratory tract infection (URTI) symptoms, stunting, cigarette smoke exposure, wood-fuelled stove, *M. catarrhalis* carriage, and *H. influenzae* carriage

^c^Adjusted for region, residence type, income, maternal education, URTI symptoms, stunting, cigarette smoke exposure, wood-fuelled stove, *S. pneumoniae* carriage, *H. influenzae* carriage, and *S. aureus* carriage

^d^Stunting (chronic undernutrition) defined as length-for-age z-score below − 2 standard deviations of the median

^e^Regional minimum salary rates (2016) were 1,800,725 Indonesian rupiah (IDR) in Padang, 2,626,940 IDR in Bandung, and 1,550,000 IDR in Lombok

^f^URTI symptoms include rhinorrhea, cough, and/or tonsillitis

In children positive for carriage, we sought to identify host and environmental factors associated with increased density using linear regression analysis. For *S. pneumoniae*, children from families in the higher income category had lower pneumococcal carriage densities, and density decreased with age (Table 3). Children with URTI symptoms or were co-colonized by either *H. influenzae* or *M. catarrhalis* had higher pneumococcal carriage densities (Table 3). For *H. influenzae*, children with two or more children under the age of five in the household had higher carriage density, whereas children whose mothers had higher education levels or who had a wood-fuelled stove in the home had lower carriage density (Table 4). For *M. catarrhalis* carriers, URTI symptoms were associated with higher density (Table 5). For *S. aureus*, children living in semi-rural areas had higher density compared to children from urban areas (Additional file 1: Table S3). When the densities of any of the four bacterial species detected were combined, URTI symptoms were positively associated with total density following multivariable analysis (Additional file 1: Table S4).

**Table 3:** Linear regression analysis of factors associated with *S. pneumoniae* density in children who are carriers (*n* = 150)

Variable (n)	Mean density (log_10_ GE/ml)^a^	Unadjusted coefficient^b^ (95% CI)	*P* value	Adjusted coefficient^c^ (95% CI)	*P* value
Region					
Padang (35)	5.09	reference	0.182		
Bandung (64)	5.17	0.08 (−0.37, 0.53)			
Lombok (51)	4.80	−0.29 (−0.76, 0.18)			
Sex					
Female (76)	4.92	reference	0.224		
Male (74)	5.14	0.22 (−0.31, 0.57)			
Residence					
Urban (73)	5.06	reference	0.761	reference	0.642
Semi-rural (77)	5.00	−0.05 (−0.41, 0.30)		−0.08 (−0.41, 0.25)	
Age (months)	5.03	−0.08 (−0.13, −0.02)	0.004	−0.06 (−0.11, −0.02)	0.009
Stunting^d^					
No (101)	4.89	reference	0.025	reference	0.142
Yes (49)	5.31	0.42 (0.06, 0.79)		0.26 (−0.09, 0.62)	
Maternal education					
Below high school (68)	5.23	reference	0.035	reference	0.360
High school and above (82)	4.86	−0.38 (−0.72, −0.03)		−0.15 (−0.47, 0.17)	
Parental monthly income^e^					
≤ regional minimum salary (100)	5.22	reference	0.003	reference	0.011
> regional minimum salary (49)	4.67	−0.56 (−0.92, −0.19)		−0.47 (−0.79, −0.10)	
Children < 5 y in the household					
1 (112)	4.97	reference	0.280		
2 or more (38)	5.19	0.22 (−0.18, 0.63)			
URTI symptoms^f^					
No (108)	4.83	reference	< 0.001	reference	0.002
Yes (42)	5.53	0.70 (0.32, 1.08)		0.55 (0.20, 0.90)	
Exposure to cigarette smoke					
No (87)	4.98	reference	0.569	reference	0.436
Yes (63)	5.09	0.10 (−0.25, 0.46)		0.13 (−0.20, 0.46)	
Wood-fuelled stove in home					
No (133)	5.07	reference	0.180	reference	0.142
Yes (17)	4.69	−0.38 (−0.93, 0.18)		−0.38 (−0.89, 0.13)	
*M. catarrhalis* carriage					
No (66)	4.70	reference	0.001	reference	0.038
Yes (84)	5.28	0.58 (0.24, 0.92)		0.33 (0.02, 0.65)	
*H. influenzae* carriage					
No (102)	4.80	reference	< 0.001	reference	0.005
Yes (48)	5.52	0.73 (0.37, 1.09)		0.49 (0.15, 0.82)	
*S. aureus* carriage					
No (142)	5.08	reference	0.019	reference	0.536
Yes (8)	4.15	−0.92 (−1.70, −0.15)		−0.22 (−0.92, 0.48)	

^a^*S. pneumoniae* density reported in log_10_ genome equivalents/ml

^b^Coefficient is the difference in means determined by linear regression

^c^Adjusted for residence type, age, maternal education, income, upper respiratory tract infection (URTI) symptoms, stunting, cigarette smoke exposure, wood-fuelled stove, *M. catarrhalis* carriage, *H. influenzae* carriage, and *S. aureus* carriage

^d^Stunting (chronic undernutrition) defined as length/height-for-age z-score below − 2 standard deviations of the median

^e^Regional minimum salary rates (2016) were 1,800,725 Indonesian rupiah (IDR) in Padang, 2,626,940 IDR in Bandung, and 1,550,000 IDR in Lombok

^f^URTI symptoms include rhinorrhea, cough, and/or tonsillitis

**Table 4:** Linear regression analysis of factors associated with *H. influenzae* density in children who are carriers (*n* = 83)

Variable (n)	Mean density (log_10_ GE/ml)^a^	Unadjusted coefficient^b^ (95% CI)	*P* value	Adjusted coefficient^c^ (95% CI)	*P* value
Region					
Padang (25)	5.51	reference	0.567		
Bandung (32)	5.67	0.16 (−0.34, 0.65)			
Lombok (26)	5.41	−0.10 (−0.62, 0.42)			
Sex					
Female (42)	5.41	reference	0.188		
Male (41)	5.68	0.27 (−0.13, 0.67)			
Residence					
Urban (39)	5.70	reference	0.152	reference	0.433
Semi-rural (44)	5.40	−0.29 (−0.70, 0.11)		−0.16 (−0.55, 0.24)	
Age (in months)	5.54	0.01 (−0.05, 0.08)	0.653		
Stunting^d^					
No (55)	5.48	reference	0.439		
Yes (28)	5.65	0.17 (−0.26, 0.60)			
Maternal education					
Below high school (38)	5.80	reference	0.020	reference	0.039
High school and above (45)	5.32	−0.47 (−0.87, −0.08)		−0.41 (−0.79, −0.02)	
Parental monthly income^e^					
≤ regional minimum salary (63)	5.62	reference	0.122	reference	0.130
> regional minimum salary (19)	5.24	−0.38 (−0.86, 0.10)		−0.34 (−0.78, 0.10)	
Children < 5y in the household					
1 (68)	5.45	reference	0.055	reference	0.021
2 or more (15)	5.96	0.51 (−0.01, 1.02)		0.59 (0.09, 1.09)	
URTI symptoms^f^					
No (61)	5.44	reference	0.118		
Yes (22)	5.81	0.36 (−0.09, 0.82)			
Exposure to cigarette smoke					
No (48)	5.46	reference	0.333	reference	0.127
Yes (35)	5.66	0.20 (−0.21, 0.61)		0.29 (−0.08, 0.67)	
Wood-fuelled stove in home					
No (74)	5.62	reference	0.020	reference	0.005
Yes (9)	4.87	−0.76 (−1.39, − 0.12)		−0.91 (−1.53, −0.28)	
*M. catarrhalis* carriage					
No (35)	5.26	reference	0.019	reference	0.053
Yes (48)	5.74	0.48 (0.08, 0.88)		0.37 (−0.01, 0.74)	
*S. pneumoniae* carriage					
No (35)	5.31	reference	0.056	reference	0.229
Yes (48)	5.71	0.39 (−0.01, 0.80)		0.23 (−0.15, 0.61)	
*S. aureus* carriage					
No (81)	5.53	reference	0.734		
Yes (2)	5.76	0.23 (−1.10, 1.56)			

^a^*H. influenzae* density reported in log_10_ genome equivalents/ml

^b^Coefficient is the difference in means determined by linear regression

^c^Adjusted for residence type, maternal education, income, 2 or more children <5y, cigarette smoke exposure, wood-fuelled stove, *M. catarrhalis* carriage, and *S. pneumoniae* carriage

^d^Stunting (chronic undernutrition) defined as length/height-for-age z-score below − 2 standard deviations of the median

^e^Regional minimum salary rates (2016) were 1,800,725 Indonesian rupiah (IDR) in Padang, 2,626,940 IDR in Bandung, and 1,550,000 IDR in Lombok

^f^Upper respiratory tract infection (URTI) symptoms include rhinorrhea, cough, and/or tonsillitis

**Table 5:** Linear regression analysis of factors associated with *M. catarrhalis* density in children who are carriers (*n* = 129)

Variable (n)	Mean density (log_10_ GE/ml)^a^	Unadjusted coefficient^b^ (95% CI)	*P* value	Adjusted coefficient^c^ (95% CI)	*P* value
Region					
Padang (32)	6.12	reference	0.010	reference	0.165
Bandung (45)	6.46	0.34 (−0.14, 0.82)		0.28 (−0.22, 0.78)	
Lombok (52)	5.81	−0.31 (−0.77, 0.15)		−0.18 (−0.68, 0.33)	
Sex					
Female (62)	6.12	reference	0.995		
Male (67)	6.12	0.00 (−0.37, 0.36)			
Residence					
Urban (57)	6.08	reference	0.748	reference	0.980
Semi-rural (72)	6.14	0.06 (−0.32, 0.44)		−0.01 (−0.41, 0.40)	
Age	6.12	−0.04 (−0.10, 0.01)	0.126		
Stunting^d^					
No (82)	6.02	reference	0.177		
Yes (47)	6.28	0.26 (−0.12, 0.65)			
Maternal education					
Below high school (65)	6.22	reference	0.258		
High school and above (64)	6.01	−0.21 (−0.58, 0.16)			
Parental monthly income^e^					
≤ regional minimum salary (92)	6.11	reference	0.885	reference	0.767
> regional minimum salary (35)	6.08	−0.03 (−0.45, 0.39)		−0.06 (−0.49, 0.36)	
Children < 5 y in the household					
1 (100)	6.06	reference	0.297		
2 or more (27)	6.30	0.24 (−0.22, 0.70)			
URTI symptoms^f^					
No (92)	5.94	reference	0.003	reference	0.035
Yes (37)	6.55	0.61 (0.21, 1.01)		0.46 (0.03, 0.88)	
Exposure to cigarette smoke					
No (69)	6.10	reference	0.863	reference	0.526
Yes (60)	6.13	0.03 (−0.34, 0.41)		0.12 (−0.27, 0.52)	
Wood-fuelled stove in home					
No (113)	6.17	reference	0.117	reference	0.534
Yes (16)	5.72	−0.45 (−1.01, 0.11)		−0.20 (−0.83, 0.43)	
*H. influenzae* carriage					
No (81)	6.05	reference	0.351		
Yes (48)	6.23	0.18 (−0.20, 0.57)			
*S. pneumoniae* carriage					
No (45)	5.87	reference	0.059	reference	0.207
Yes (84)	6.25	0.37 (−0.01, 0.76)		0.25 (−0.14, 0.65)	
*S. aureus* carriage					
No (128)	6.11	reference	0.236		
Yes (1)	7.38	1.27 (−0.84, 3.39)			

^a^*M. cattarhalis* density reported in log_10_ genome equivalents/ml

^b^Coefficient is the difference in means determined by linear regression

^c^Adjusted for region, residence type, income, upper respiratory tract infection (URTI) symptoms, cigarette smoke exposure, wood-fuelled stove, and *S. pneumoniae* carriage

^d^Stunting (chronic undernutrition) defined as length/height-for-age z-score below −2 standard deviations of the median

^e^Regional minimum salary rates (2016) were 1,800,725 Indonesian rupiah (IDR) in Padang, 2,626,940 IDR in Bandung, and 1,550,000 IDR in Lombok

^f^URTI symptoms include rhinorrhea, cough, and/or tonsillitis

## Discussion

Using data from a cross-sectional study, we identified risk factors associated with carriage and/or density of four clinically-relevant bacterial species in Indonesian children. Contrary to expectation, we did not observe major differences in carriage of these bacterial species between children living in urban and semi-rural environments, although semi-rural children had higher densities of *S. aureus* compared to urban children. Of the potential risk factors assessed, the majority did not differ between these two groups. The semi-rural sites involved in our study were located within an hour drive of the urban areas to enable timely storage of samples. It is possible that children in more remote rural areas may have different characteristics and increased risk of bacterial carriage than the children assessed in our study. We also only included sites from three of Indonesia’s 34 provinces. HIV status was not determined for participants in our study, but HIV prevalence in Indonesian children is likely quite low, as overall prevalence of HIV in Indonesia is estimated to be < 0.5% [35].

The risk factors for *S. pneumoniae* carriage in our study, the presence of two or more children under the age of five years in the household, and having URTI symptoms, were consistent with studies conducted on children of a similar age in other countries [10, 11]. Having URTI symptoms was also identified as risk factors for *M. catarrhalis* carriage. We did not identify any epidemiologic factors significantly associated with carriage of *H. influenzae* or *S. aureus*. Interactions between bacterial species may also affect carriage dynamics, and we previously reported positive relationships among *S. pneumoniae*, *H. influenzae*, and *M. catarrhalis* [28]. Using multivariable models that accounted for bacterial and epidemiologic factors, carriage of *M. catarrhalis* was a risk factor for *S. pneumoniae* and *H. influenzae* carriage, but negatively associated with *S. aureus* carriage, and vice versa. These relationships are consistent with the literature, and suggest that both intrinsic host factors as well as co-colonizing bacteria influence the nasopharyngeal microbiome [7, 8].

Bacterial density in the nasopharynx is linked to both the development of infection and pathogen transmission. In a study in Vietnamese children under five years old, pneumococcal loads in the nasopharynx were significantly higher in children with radiologically confirmed pneumonia compared to children with other lower respiratory tract infection or healthy controls [22]. However, relatively few data on risk factors associated with bacterial density are available. In our study, we identified host, socio-economic, and environmental factors associated with increased bacterial density in the nasopharynx, although results differed depending on the bacterial species. For *S. pneumoniae*, low parental income, URTI symptoms, and co-colonization with *M. catarrhalis* or *H. influenzae* were associated with increased density, whereas density decreased with age. Declining pneumococcal density with age has been reported previously [36]. URTI symptoms were associated with increased density of *S. pneumoniae* and *M. catarrhalis.* It is unclear whether the presence of these species at high density may be causative of the observed symptoms, the presence of respiratory symptoms creates an environment that favors bacterial growth, or both.

A limitation of our study is that respiratory viruses were not assessed. There is mounting evidence that they play a role in pneumococcal carriage density. A study conducted on children in day-care in Portugal found that pneumococcal colonization was associated with rhinitis symptoms, consistent with our findings, and pneumococcal density was significantly higher in children who tested positive for a respiratory virus [25]. In a randomized trial of live attenuated influenza vaccine (LAIV) in the UK, children who received the vaccine had significantly higher pneumococcal density in the nasopharynx 28 days after vaccination compared to controls [37]. In rural children under the age of three in Peru, pneumococcal densities were higher during acute respiratory illness (*p* < 0.0001), and higher in children who tested positive for a respiratory virus compared to those who were virus-negative. (median log_10_ transformed density 4.73 vs 3.94, respectively; adjusted *p* = 0.06) [38]. Based upon these findings, it is likely that some children in our study who had URTI symptoms and high pneumococcal density may have been co-infected with a respiratory virus. A recent study in American children aged 4–7 years reported that pneumococcal densities were higher when a respiratory virus was detected, regardless of whether the children displayed URTI symptoms [39]. These data suggest that respiratory viruses may influence bacterial density in asymptomatic children as well as those experiencing respiratory infections. Viral testing is recommended for future studies on risk factors for bacterial carriage.

For *M. catarrhalis*, there are few published data examining density, however children in Tanzania with severe pneumonia had increased density of *M. catarrhalis* in their nasopharynx compared to children with mild URTI [40]. In our study, we did not find an association between *H. influenzae* density and URTI symptoms. In the Portuguese day care study, rhinitis symptoms were associated with increased *H. influenzae* density, but in the LAIV trial, vaccine recipients did not have higher *H. influenzae* density compared to controls [25, 37]. The relationship between *H. influenzae* density and URTI symptoms may depend on the study population and/or respiratory virus.

We sought to examine the effects of smoke exposure on carriage of potentially pathogenic bacteria by including exposure to cigarette smoke and the presence of wood-fuelled stoves in the home as variables. Unlike a study on children aged 1–59 months in Israel, and a study that included children aged 6–60 months and 45–70 year old adults in Semarang, Indonesia, we did not find an association between pneumococcal carriage and cigarette smoke exposure [29, 41]. However, studies on children and teenagers conducted in the Netherlands and the UK similarly did not find an association with cigarette smoke exposure, nor did two earlier studies conducted in Lombok, Indonesia [30, 31, 42, 43]. Levels of smoke exposure are difficult to ascertain without detailed monitoring, and our analysis did not incorporate whether the smoker in the household was the mother, father, or other relative. Wood-fuelled stoves, which can contribute to indoor air pollution and are associated with increased risk of pneumonia, likewise were not a risk factor for bacterial carriage in our study [44]. Unexpectedly, the presence of wood-fuelled stoves was associated with decreased density of *H. influenzae* and *S. aureus*. Most Indonesian households use liquefied petroleum gas (LPG) stoves as LPG is subsidized by the government. Relatively few participants in our study reported having a wood-fuelled stove, and some of those who did also had an LPG stove. Due to the small numbers these results should be interpreted with caution.

The high prevalence (26.8%) of stunting, an indicator of chronic undernutrition, in our study was consistent with 2013 national data from Indonesia showing stunting in 36.4% of children under the age of five (https://data.worldbank.org/) and a 2011 study reporting 28.4% stunting in children under 24 months [45]. Stunting is a recognized risk factor for pneumonia in children, as well as for poor outcomes in pediatric pneumonia [46, 47]. Recently, stunting was identified as a risk factor for *S. pneumoniae* carriage in Warao Amerindians in Venezuela aged 0–4 years [48]. In our study, stunting was associated with increased odds of carriage for *S. pneumoniae* and *M. catarrhalis*, and increased *S. pneumoniae* density in univariable analysis, but these associations were no longer significant for *S. pneumoniae* following adjustment. However, taken together, data suggest that children with chronic malnutrition are more susceptible to colonization and/or high density carriage by potentially pathogenic bacteria, which may increase their risk of bacterial infections. Interventions to improve childhood nutrition as a potential strategy to reduce infections in at-risk populations may be worthy of investigation.

Low maternal education and having two or more children under five years in the household were associated with increased density of *H. influenzae*. These data, along with the association between low family income and higher density of *S. pneumoniae*, suggest that factors related to socio-economic status and exposure (interaction with other young children) may increase carriage density of some pathogens in addition to increasing risk of carriage. To our knowledge, these are the first published data linking socio-economic factors to bacterial carriage density.

## Conclusions

Our study findings highlight the importance of socio-economic factors, such as maternal education levels and having multiple young children in the household, as risk factors for carriage of potentially pathogenic bacteria. Stunting, a recognized public health problem in Indonesia, was linked to increased pathogen carriage and density. These factors are indicative of poverty, as are the majority of risk factors associated with pneumonia in children [2]. Our results provide further data demonstrating the association between URTIs and pathogen presence and density in the nasopharynx. There is growing evidence that bacterial density in the nasopharynx can play a role in development of pneumonia and other infections, as well as pathogen transmission. Here, we provide novel data on host and environmental factors associated with bacterial density.

## Additional file

Additional file 1: **Table S1.** Univariable and multivariable analysis of risk factors for H. influenzae and S.aureus carriage. **Table S2.** Univariable and multivariable analysis of risk factors for carriage of two or more of the following species: *S. pneumoniae*, *H. influenzae*, *M. catarrhalis*, and *S. aureus*. **Table S3.** Linear regression analysis of factors associated with *S. aureus* density in children who are carriers (*n* = 22). **Table S4.** Linear regression analysis of factors associated with the combined density of *S. pneumoniae*, *H. influenzae*, *M. catarrhalis*, and/or *S. aureus* in children colonized with of two or more bacterial species (*n* = 125). (DOCX 50 kb)

## Abbreviations

aOR: adjusted odds ratio
CI: Confidence intervals
GE: Genome equivalents
Hib: *Haemophilus influenzae* type B
HIV: Human immunodeficiency virus
IDR: Indonesian rupiah
LAIV: Live attenuated influenza vaccine
LPG: Liquid petroleum gas
OR: Odds ratio
PCR: Polymerase chain reaction
PCV: Pneumococcal conjugate vaccine
qPCR: real-time quantitative PCR
UK: United Kingdom
URTI: Upper respiratory tract infection
WHO: World Health Organization

## References

[1] Liu L, Oza S, Hogan D, Chu Y, Perin J, Zhu J (2016). Global, regional, and national causes of under-5 mortality in 2000–15: an updated systematic analysis with implications for the sustainable development goals. Lancet.

[2] Rudan I, Boschi-Pinto C, Biloglav Z, Mulholland K, Campbell H (2008). Epidemiology and etiology of childhood pneumonia. Bull World Health Organ.

[3] Murphy TF, Bakaletz LO, Smeesters PR (2009). Microbial interactions in the respiratory tract. Pediatr Infect Dis J.

[4] Bogaert D, de Groot R, Hermans PWM (2004). *Streptococcus pneumoniae* colonisation: the key to pneumococcal disease. Lancet Infect Dis.

[5] Adegbola RA, DeAntonio R, Hill PC, Roca A, Usuf E, Hoet B (2014). Carriage of *Streptococcus pneumoniae* and other respiratory bacterial pathogens in low and lower-middle income countries: a systematic review and meta-analysis. PLoS One.

[6] Watson K, Carville K, Bowman J, Jacoby P, Riley TV, Leach AJ. Upper respiratory tract bacterial carriage in Aboriginal and non-Aboriginal children in a semi-arid area of Western Australia. Pediatr Infect Dis J. 2006 Sep;25(9):782–90. 10.1097/01.inf.0000232705.49634.68 16940834

[7] Dunne EM, Manning J, Russell FM, Robins-Browne RM, Mulholland EK, Satzke C (2012). Effect of pneumococcal vaccination on nasopharyngeal carriage of *Streptococcus pneumoniae*, *Haemophilus influenzae*, *Moraxella catarrhalis*, and *Staphylococcus aureus* in Fijian children. J Clin Microbiol.

[8] Kwambana B, Barer M, Bottomley C, Adegbola R, Antonio M (2011). Early acquisition and high nasopharyngeal co-colonisation by *Streptococcus pneumoniae* and three respiratory pathogens amongst Gambian new-borns and infants. BMC Infect Dis.

[9] Regev-Yochay G, Raz M, Dagan R, Porat N, Shainberg B, Pinco E (2004). Nasopharyngeal carriage of *Streptococcus pneumoniae* by adults and children in community and family settings. Clin Infect Dis.

[10] Toledo ME, Casanova MF, Linares-Pérez N, García-Rivera D, Toraño Peraza G, Barcos Pina I (2017). Prevalence of pneumococcal nasopharyngeal carriage among. Children 2–18 months of age: baseline study pre introduction of pneumococcal vaccination in Cuba. Pediatr Infect Dis J.

[11] Abdullahi O, Nyiro J, Lewa P, Slack M, Scott JAG. The descriptive epidemiology of *Streptococcus pneumoniae* and *Haemophilus influenzae* nasopharyngeal carriage in children and adults in Kilifi District, Kenya. Pediatr Infect Dis J. 2008;27. 10.1097/INF.0b013e31814da70c PMC2382474 18162940

[12] Labout JAM, Duijts L, Arends LR, Jaddoe VWV, Hofman A, de Groot R (2008). Factors associated with pneumococcal carriage in healthy Dutch infants: the Generation R Study. J Pediatr.

[13] Jourdain S, Smeesters PR, Denis O, Dramaix M, Sputael V, Malaviolle X, Van Melderen L, Vergison A (2011). Differences in nasopharyngeal bacterial carriage in preschool children from different socio-economic origins. Clin Microbiol Infect.

[14] Principi N, Marchisio P, Schito GC, Mannelli S (1999). Ascanius project collaborative group. Risk factors for carriage of respiratory pathogens in the nasopharynx of healthy children. Pediatr Infect Dis J.

[15] Hanieh S, Hamaluba M, Kelly DF, Metz JA, Wyres KL, Fisher R (2014). *Streptococcus pneumoniae* carriage prevalence in Nepal: evaluation of a method for delayed transport of samples from remote regions and implications for vaccine implementation. PLoS One.

[16] Neto AS, Lavado P, Flores P, Dias R, Pessanha MA, Sousa E (2003). Risk factors for the nasopharyngeal carriage of respiratory pathogens by Portuguese children: phenotype and antimicrobial susceptibility of *Haemophilus influenzae* and *Streptococcus pneumoniae*. Microb Drug Resist.

[17] Kinabo GD, Ven A, Msuya LJ, Shayo AM, Schimana W, Ndaro A (2013). Dynamics of nasopharyngeal bacterial colonisation in HIV-exposed young infants in Tanzania. Tropical Med Int Health.

[18] Verani JR, Massora S, Acácio S, dos Santos RT, Vubil D, Pimenta F, et al. Nasopharyngeal carriage of *Streptococcus pneumoniae* among HIV-infected and –uninfected children <5 years of age before introduction of pneumococcal conjugate vaccine in Mozambique. PLoS One. 2018;13:e0191113. 10.1371/journal.pone.0191113 PMC5813901 29447196

[19] Madhi SA, Adrian P, Kuwanda L, Cutland C, Albrich WC, Klugman KP (2007). Long-term effect of pneumococcal conjugate vaccine on nasopharyngeal colonization by *Streptococcus pneumoniae*—and associated interactions with *Staphylococcus aureus* and *Haemophilus influenzae* colonization—in HIV-infected and HIV-uninfected children. J Infect Dis.

[20] Donkor ES, Annan JA, Badoe EV, Dayie NTKD, Labi A-K, Slotved H-C. Pneumococcal carriage among HIV infected children in Accra, Ghana. BMC Infect Dis. 2017;17:133. 10.1186/s12879-017-2224-0 PMC5299768 28178935

[21] Mulu W, Yizengaw E, Alemu M, Mekonnen D, Hailu D, Ketemaw K, et al. Pharyngeal colonization and drug resistance profiles of *Morraxella catarrrhalis*, *Streptococcus pneumoniae*, *Staphylococcus aureus*, and *Haemophilus influenzae* among HIV infected children attending ART Clinic of Felegehiwot referral hospital, Ethiopia. PloS One. 2018;13:e0196722. 10.1371/journal.pone.0196722 PMC5944927 29746496

[22] Vu HTT, Yoshida LM, Suzuki M, Nguyen HAT, Nguyen CDL, Nguyen ATT (2011). Association between nasopharyngeal load of *Streptococcus pneumoniae*, viral coinfection, and radiologically confirmed pneumonia in Vietnamese children. Pediatr Infect Dis J.

[23] Short KR, Reading PC, Wang N, Diavatopoulos DA, Wijburg OL. Increased nasopharyngeal bacterial titers and local inflammation facilitate transmission of *Streptococcus pneumoniae*. MBio. 2012;3(5):e00255-12.. 10.1128/mBio.00255-12 PMC3518912 23015738

[24] Binks M, Cheng A, Smith-Vaughan H, Sloots T, Nissen M, Whiley D (2011). Viral-bacterial co-infection in Australian indigenous children with acute otitis media. BMC Infect Dis.

[25] Rodrigues F, Foster D, Nicoli E, Trotter C, Vipond B, Muir P (2013). Relationships between rhinitis symptoms, respiratory viral infections and nasopharyngeal colonization with *Streptococcus pneumoniae*, *Haemophilus influenzae* and *Staphylococcus aureus* in children attending daycare. Pediatr Infect Dis J.

[26] UNICEF (2014). Committing to child survival: a promise renewed.

[27] Hodge A, Firth S, Marthias T, Jimenez-Soto E. Location Matters: Trends in inequalities in child mortality in Indonesia. Evidence from repeated cross-sectional surveys. PLoS One. 2014;9(7):e103597. 10.1371/journal.pone.0103597 PMC4111602 25061950

[28] Dunne EM, Murad C, Sudigdoadi S, Fadlyana E, Tarigan R, Indriyani SAK, Pell CL, Watts E, Satzke C, Hinds J (2018). Carriage of *Streptococcus pneumoniae*, *Haemophilus influenzae*, *Moraxella catarrhalis*, and *Staphylococcus aureus* in Indonesian children: a cross-sectional study. PLoS One.

[29] Farida H, Severin JA, Gasem MH, Keuter M, Wahyono H, van den Broek P (2014). Nasopharyngeal carriage of *Streptococcus pneumonia* in pneumonia-prone age groups in Semarang, Java Island, Indonesia. PLoS One.

[30] Soewignjo S, Gessner BD, Sutanto A, Steinhoff M, Prijanto M, Nelson C, et al. *Streptococcus pneumoniae* nasopharyngeal carriage prevalence, serotype distribution, and resistance patterns among children on Lombok Island. Indonesia Clin Infect Dis. 2001;32(7):1039–43. 10.1086/319605 11264032

[31] Hadinegoro SR, Prayitno A, Khoeri MM, Djelantik IG, Dewi NE, Indriyani SA (2016). Nasopharyngeal carriage of *Streptococcus pneumoniae* in healthy children under five years old in Central Lombok regency, Indonesia. Southeast Asian J Trop Med Public Health.

[32] Mercedes O (2006). WHO child growth standards based on length/height, weight and age. Acta Paediatr.

[33] Satzke C, Turner P, Virolainen-Julkunen A, Adrian PV, Antonio M, Hare KM (2013). Standard method for detecting upper respiratory carriage of *Streptococcus pneumoniae*: updated recommendations from the World Health Organization pneumococcal carriage working group. Vaccine.

[34] Carvalho Mda G, Tondella ML, McCaustland K, Weidlich L, McGee L, Mayer LW (2007). Evaluation and improvement of real-time PCR assays targeting *lytA*, *ply*, and *psaA* genes for detection of pneumococcal DNA. J Clin Microbiol.

[35] Januraga PP, Reekie J, Mulyani T, Lestari BW, Iskandar S, Wisaksana R (2018). The cascade of HIV care among key populations in Indonesia: a prospective cohort study. Lancet HIV.

[36] Roca A, Bottomley C, Hill PC, Bojang A, Egere U, Antonio M (2012). Effect of age and vaccination with a pneumococcal conjugate vaccine on the density of pneumococcal nasopharyngeal carriage. Clin Infect Dis.

[37] Thors V, Christensen H, Morales-Aza B, Vipond I, Muir P, Finn A (2016). The effects of live attenuated influenza vaccine on nasopharyngeal bacteria in healthy 2 to 4 year olds. A randomized controlled trial. Am J Respir Crit Care Med.

[38] Fan RR, Howard LM, Griffin MR, Edwards KM, Zhu Y, Williams JV (2016). Nasopharyngeal pneumococcal density and evolution of acute respiratory illnesses in young children, Peru, 2009–2011. Emerg Infect Dis.

[39] DeMuri GP, Gern JE, Eickhoff JC, Lynch SV, Wald ER (2018). Dynamics of bacterial colonization with *Streptococcus pneumoniae*, *Haemophilus influenzae*, and *Moraxella catarrhalis* during symptomatic and asymptomatic viral upper respiratory tract infection. Clin Infect Dis.

[40] Chochua S, D'Acremont V, Hanke C, Alfa D, Shak J, Kilowoko M (2016). Increased nasopharyngeal density and concurrent carriage of *Streptococcus pneumoniae*, *Haemophilus influenzae*, and *Moraxella catarrhalis* are associated with pneumonia in febrile children. PLoS One.

[41] Greenberg D, Givon-Lavi N, Broides A, Blancovich I, Peled N, Dagan R (2006). The contribution of smoking and exposure to tobacco smoke to *Streptococcus pneumoniae* and *Haemophilus influenzae* carriage in children and their mothers. Clin Infect Dis.

[42] van Hoek AJ, Sheppard CL, Andrews NJ, Waight PA, Slack MPE, Harrison TG (2014). Pneumococcal carriage in children and adults two years after introduction of the thirteen valent pneumococcal conjugate vaccine in England. Vaccine.

[43] Bogaert D, van Belkum A, Sluijter M, Luijendijk A, de Groot R, Rümke HC (2004). Colonisation by *Streptococcus pneumoniae* and *Staphylococcus aureus* in healthy children. Lancet.

[44] Dherani M, Pope D, Mascarenhas M, Smith KR, Weber M, Bruce N (2008). Indoor air pollution from unprocessed solid fuel use and pneumonia risk in children aged under five years: a systematic review and meta-analysis. Bull World Health Organ.

[45] Torlesse H, Cronin AA, Sebayang SK, Nandy R (2016). Determinants of stunting in Indonesian children: evidence from a cross-sectional survey indicate a prominent role for the water, sanitation and hygiene sector in stunting reduction. BMC Public Health.

[46] Moschovis PP, Addo-Yobo EO, Banajeh S, Chisaka N, Christiani DC, Hayden D (2015). Stunting is associated with poor outcomes in childhood pneumonia. Tropical Med Int Health.

[47] Schlaudecker EP, Steinhoff MC, Moore SR (2011). Interactions of diarrhea, pneumonia, and malnutrition in childhood: recent evidence from developing countries. Curr Opin Infect Dis.

[48] Verhagen LM, Hermsen M, Rivera-Olivero IA, Sisco MC, de Jonge MI, Hermans PW (2017). Nasopharyngeal carriage of respiratory pathogens in Warao Amerindians: significant relationship with stunting. Tropical Med Int Health.

