# Supplementary material for: Risk factors associated with nasopharyngeal carriage and density of Streptococcus pneumoniae, Haemophilus influenzae, Moraxella catarrhalis, and Staphylococcus aureus in young children living in Indonesia
Source: Pneumonia (Nathan). 2018 Dec 25;10:14. doi: 10.1186/s41479-018-0058-1 (PMC6305570; doi:10.1186/s41479-018-0058-1)
Supplement: Supplementary file 1 — Table S1. Univariable and multivariable analysis of risk factors for H. influenzae and S.aureus carriage. Table S2. Univariable and multivariable analysis of risk factors for carriage of two or more of the following species: S. pneumoniae, H. influenzae, M. catarrhalis, and S. aureus. Table S3. Linear regression analysis of factors associated with S. aureus density in children who are carriers (n = 22). Table S4. Linear regression analysis of factors associated with the combined density of S. pneumoniae, H. influenzae, M. catarrhalis, and/or S. aureus in children colonized with of two or more bacterial species (n = 125). (DOCX 50 kb) [file 41479_2018_58_MOESM1_ESM.docx]

**Additional file 1**

**Detection of bacterial pathogens by qPCR**

Pneumococci were detected by singleplex real-time quantitative PCR (qPCR) targeting the *lytA* gene [1]. Primer and probe sequences (5’ to 3’) were as follows: forward primer CGCAATCTAGCAGATGAAGCA, reverse primer TCGTGCGTTTTAATTCCAGCT, probe TGCCGAAAACGCTTGATACAGGGAG (5' FAM; 3' BHQ1). qPCR was conducted in 25 μl reactions containing 5 μl of template DNA, 200nM of each primer and probe, and 1X TaqMan GeneExpression Mastermix (Applied Biosystems). qPCR was conducted using an Applied Biosystems 7500 real-time PCR machine and the following cycling conditions: 95°C for 10 min followed by 40 cycles of: 95°C for 15 sec and 60°C for 1 min.

*H. influenzae*, *M. catarrhalis*, and *S. aureus* were detected by multiplex qPCR using the FTD Bacterial Pneumonia CAP qPCR kit (Fast-Track Diagnostics). qPCR was conducted in 25 μl reactions containing 10 μl of template DNA using buffer, enzyme, and primer probe mix provided in the kit according to the manufacturer’s instructions. Reactions were run on an Applied Biosystems 7500 real-time PCR machine using the following cycling conditions: 42°C for 15 min, then 94ºC for 3 min, followed by 40 cycles of 94°C for 8 seconds and 60°C for 34 seconds.

For both assays, standard curves for quantification were prepared using plasmid standards containing a single copy of the target gene (Fast-Track Diagnostics). Standards and no template controls were run in triplicate wells, whereas samples and extraction controls were run in singlicate. Results were reported as genome equivalents/ml (GE/ml), which estimates bacterial density assuming each bacterial cell contains one genome with a single copy of the target gene. For *H. influenzae*, *M. catarrhalis*, and *S. aureus*, samples with a Ct value < 35 were considered positive and 35–40 considered negative. For pneumococcus, samples with a Ct value < 35 were considered positive, and those from 35–40 considered equivocal and confirmed by culture. Limits of detection were 40 GE/ml for *S. pneumoniae*, 3,890 GE/ml for *H. influenzae*, 8,700 GE/ml for *M. catarrhalis*, and 3,570 GE/ml for *S. aureus*.

**Table S1. Univariable and multivariable analysis of risk factors for *H. influenzae* and *S.aureus* carriage**

|  | | ***H. influenzae*** | | | | | ***S. aureus*** | | | | |  |  |
| --- | --- | --- | --- | --- | --- | --- | --- | --- | --- | --- | --- | --- | --- |
| **Variable** | | **carriers/total (%)** | **Unadjusted OR^1^ (95% CI)** | **P value** | **Adjusted OR^2^**  **(95% CI)** | **P value** | **carriers/total (%)** | **Unadjusted OR (95% CI)** | **P value** | **Adjusted OR^3^**  **(95% CI)** | **P value** |  |  |
| **Region** | | | | | | | | | | |  |  |  |
|  | Padang | 25/101 (24.8) | reference | 0.461 |  |  | 8/101 (7.9) | reference | 0.956 |  |  |  |  |
|  | Bandung | 32/100 (32.0) | 1.14 (0.77, 2.65) |  |  |  | 7/100 (7.0) | 0.88 (0.30, 2.51) |  |  |  |  |  |
|  | Lombok | 26/101 (25.8) | 1.05 (0.56, 1.99) |  |  |  | 7/101 (6.9) | 0.87 (0.30, 2.48) |  |  |  |  |  |
| **Sex** | | | | | | | | | | |  |  |  |
|  | Female | 42/144 (29.2) | reference | 0.532 |  |  | 7/144 (4.9) | reference | 0.128 |  |  |  |  |
|  | Male | 41/158 (26.0) | 0.85 (0.51, 1.41) |  |  |  | 15/158 (9.5) | 2.05 (0.81, 5.19) |  |  |  |  |  |
| **Residence** | | | | | | | | | | |  |  |  |
|  | Urban | 39/152 (25.7) | reference | 0.475 | reference | 0.610 | 14/152 (9.2) | reference | 0.200 | reference | 0.440 |  |  |
|  | Semi-rural | 44/150 (29.3) | 1.20 (0.72, 1.99) |  | 1.15 (0.66, 2.00) |  | 8/150 (5.3) | 0.56 (0.23, 1.36) |  | 0.68 (0.25, 1.83) |  |  |  |
| **Age (months)** | |  | 0.98 (0.91, 1.06) | 0.593 |  |  |  | 1.08 (0.94, 1.24) | 0.251 |  |  |  |  |
| **Stunting^4^** | | | | | | | | | | |  |  |  |
|  | No | 55/221 (24.9) | reference | 0.096 | reference | 0.508 | 18/221 (8.1) | reference | 0.347 |  |  |  |  |
|  | Yes | 28/81 (34.6) | 1.60 (0.92, 2.76) |  | 1.22 (0.68, 2.20) |  | 4/81 (4.9) | 0.56 (0.19, 1.79) |  |  |  |  |  |
| **Maternal education** | | | | | | | | | | |  |  |  |
|  | Below high school | 38/119 (31.9) | reference | 0.163 |  |  | 5/119 (4.2) | reference | 0.105 |  |  |  |  |
|  | High school and above | 45/183 (24.6) | 0.70 (0.42, 1.16) |  |  |  | 17/183 (9.3) | 2.34 (0.84, 6.51) |  |  |  |  |  |
| **Parental monthly income^5^** | | | | | | | | | | | |  |  |
|  | ≤ Regional minimum salary | 63/210 (30.0) | reference | 0.115 | reference | 0.182 | 11/210 (5.2) | reference | 0.039 | reference | 0.082 |  |  |
|  | > Regional minimum salary | 19/90 (21.1) | 0.62 (0.35, 1.12) |  | 0.66 (0.36, 1.21) |  | 11/90 (12.2) | 2.52 (1.05, 6.04) |  | 2.29 (0.90, 5.83) |  |  |  |
| **Children <5 y in the household** | | | | | | | | | | | |  |  |
|  | 1 | 68/237 (28.7) | reference | 0.482 |  |  | 21/237 (8.9) | reference | 0.085 | reference | 0.079 |  |  |
|  | 2 or more | 15/62 (24.2) | 0.79 (0.42, 1.51) |  |  |  | 1/62 (1.6) | 0.17 (0.02, 1.28) |  | 0.16 (0.02, 1.24) |  |  |  |
| **URTI symptoms^6^** | | | | | | | | | | | |  |  |
|  | No | 61/240 (25.4) | reference | 0.115 |  |  | 20/240 (8.3) | reference | 0.184 |  |  |  |  |
|  | Yes | 22/62 (35.5) | 1.61 (0.89, 2.93) |  |  |  | 2/62 (3.2) | 0.37 (0.08, 1.61) |  |  |  |  |  |
| **Exposure to cigarette smoke** | | | | | | | | | | | |  |  |
|  | No | 48/163 (29.4) | reference | 0.408 | reference | 0.456 | 13/163 (8.0) | reference | 0.618 | reference | 0.974 |  |  |
|  | Yes | 35/139 (25.2) | 0.81 (0.48, 1.34) |  | 0.81 (0.47, 1.40) |  | 9/139 (6.5) | 0.80 (0.33, 1.93) |  | 0.98 (0.37, 2.60) |  |  |  |
| **Wood-fuelled stove in home** | | | | | | | | | | | |  |  |
|  | No | 74/268 (27.6) | reference | 0.888 | reference | 0.783 | 113/268 (42.2) | reference | 0.587 | reference | 0.738 |  |  |
|  | Yes | 9/34 (26.5) | 0.94 (0.42, 2.12) |  | 0.89 (0.37, 2.10) |  | 16/34 (47.1) | 1.23 (0.60, 2.50) |  | 1.33 (0.25, 6.96) |  |  |  |
| ***M. catarrhalis* carriage** | | |  |  |  |  |  |  |  |  |  |  |  |
|  | No | 35/173 (20.2) | reference | 0.001 | reference | 0.023 | 21/152 (12.1) | reference | 0.005 | reference | 0.010 |  |  |
|  | Yes | 48/129 (37.2) | 2.34 (1.40, 3.91) |  | 1.90 (1.09, 3.30) |  | 1/150 (0.8) | 0.06 (0.01, 0.43) |  | 0.07 (0.01, 0.53) |  |  |  |
| ***S. pneumoniae* carriage** | | |  |  |  |  |  |  |  |  |  |  |  |
|  | No | 35/152(23.0) | reference | 0.082 | reference | 0.311 | 18/219 (9.2) | reference | 0.200 |  |  |  |  |
|  | Yes | 48/150 (32.0) | 1.57 (0.94, 2.62) |  | 1.33 (0.77, 2.30) |  | 8/83 (5.3) | 0.56 (0.23, 1.36) |  |  |  |  |  |
| ***S. aureus* carriage** | |  |  |  |  |  | ***H. influenzae* carriage** | |  |  |  |  |  |
|  | No | 81/280 (28.9) | reference | 0.062 | reference | 0.205 | 20/280 (9.1) | reference | 0.062 | reference | 0.184 |  |  |
|  | Yes | 2/22 (9.1) | 0.25 (0.06, 1.08) |  | 0.38 (0.08, 1.71) |  | 222 (2.4) | 0.25 (0.06, 1.08) |  | 0.36 (0.08, 1.63) |  |  |  |

^1^OR = odds ratio

^2^Adjusted for residence type, stunting, income, cigarette smoke exposure, wood-fuelled stove, *M. catarrhalis* carriage, *S. pneumoniae* carriage, and *S. aureus* carriage

^3^Adjusted for residence type, income, 2 or more children <5y, cigarette smoke exposure, wood-fuelled stove, *M. catarrhalis* carriage, and *H. influenzae* carriage

^4^Stunting (chronic undernutrition) defined as length-for-age Z score below -2

^5^Regional minimum salary rates (2016) were 1,800,725 Indonesian rupiah (IDR) in Padang, 2,626,940 IDR in Bandung, and 1,550,000 IDR in Lombok

^6^URTI symptoms include rhinorrhea, cough, and/or tonsillitis

**Table S2. Univariable and multivariable analysis of risk factors for carriage of two or more of the following species: *S. pneumoniae*, *H. influenzae*, *M. catarrhalis*, and *S. aureus*.**

|  | | **Multiple species carriage** | | | | |
| --- | --- | --- | --- | --- | --- | --- |
| **Variable** | | **Multiple species carriers/total (%)** | **Unadjusted OR^1^ (95% CI)** | **P value** | **Adjusted OR^2^**  **(95% CI)** | **P value** |
| **Region** | | | | | | |
|  | Padang | 23/101 (22.8) | reference | <0.001 | reference | 0.001 |
|  | Bandung | 54/100 (54.0) | 3.98 (2.16, 7.32) |  | 3.20 (1.59, 6.44) |  |
|  | Lombok | 48/101 (47.5) | 3.07 (1.67, 5.64) |  | 3.22 (1.62, 6.38) |  |
| **Sex** | | | | | | |
|  | Female | 59/144 (41.0) | reference | 0.888 |  |  |
|  | Male | 66/158 (41.8) | 1.03 (0.65, 1.63) |  |  |  |
| **Residence** | | | | | | |
|  | Urban | 60/152 (39.5) | reference | 0.496 | reference | 0.923 |
|  | Semi-rural | 65/150 (43.3) | 1.17 (0.74, 1.85) |  | 1.03 (0.61, 1.74) |  |
| **Age (months)** | |  | 0.99 (0.92, 1.06) | 0.709 |  |  |
| **Stunting^3^** | | | | | | |
|  | No | 80/221 (36.2) | reference | 0.003 | reference | 0.241 |
|  | Yes | 45/81 (55.6) | 2.20 (1.31, 3.69) |  | 1.42 (0.79, 2.57) |  |
| **Maternal education** | | | | | | |
|  | Below high school | 58/119 (48.7) | reference | 0.037 | reference | 0.354 |
|  | High school and above | 67/183 (36.6) | 0.61 (0.38, 0.97) |  | 0.77 (0.45, 1.33) |  |
| **Parental monthly income^4^** | | | | | | |
|  | ≤ Regional minimum salary | 83/210 (39.5) | reference | 0.743 | reference | 0.709 |
|  | > Regional minimum salary | 40/90 (44.4) | 1.22 (0.74, 2.02) |  | 1.12 (0.62 2.01) |  |
| **Children <5 y in the household** | | | | | | |
|  | 1 | 101/237 (42.6) | reference | 0.579 |  |  |
|  | 2 or more | 24/62 (38.7) | 0.85 (0.48, 1.51) |  |  |  |
| **URTI symptoms^5^** | | | | | | |
|  | No | 86/240 (35.8) | reference | <0.001 | reference | <0.001 |
|  | Yes | 39/62 (62.9) | 3.04 (1.71, 5.42) |  | 3.21 (1.69, 6.11) |  |
| **Exposure to cigarette smoke** | | | | | | |
|  | No | 75/163 (46.0) | reference | 0.078 | reference | 0.509 |
|  | Yes | 50/139 (36.0) | 0.66 (0.42, 1.05) |  | 0.84 (0.50, 1.41) |  |
| **Wood-fuelled stove in home** | | | | | | |
|  | No | 111/268 (41.4) | reference | 0.979 | reference | 0.629 |
|  | Yes | 14/34 (41.2) | 0.99 (0.48, 2.04) |  | 1.22 (0.54, 2.79) |  |

^1^OR = odds ratio

^2^Adjusted for region, residence type, stunting, maternal education, income, upper respiratory tract infection (URTI) symptoms, cigarette smoke exposure, and wood-fuelled stove.

^3^Stunting (chronic undernutrition) defined as length-for-age Z score below -2

^4^Regional minimum salary rates (2016) were 1,800,725 Indonesian rupiah (IDR) in Padang, 2,626,940 IDR in Bandung, and 1,550,000 IDR in Lombok

^5^URTI symptoms include rhinorrhea, cough, and/or tonsillitis

**Table S3. Linear regression analysis of factors associated with *S. aureus* density in children who are carriers (n=22).**

| **Variable (n)** | | **Mean density (log_10_ GE/ml)^1^** | **Unadjusted coefficient^2^ (95% CI)** | **P value** | **Adjusted coefficient^3^**  **(95% CI)** | **P value** |
| --- | --- | --- | --- | --- | --- | --- |
| **Region** | | | | | | |
|  | Padang (8) | 4.50 | reference | 0.119 |  |  |
|  | Bandung (7) | 5.48 | 0.97 (-0.13, 2.08) |  |  |  |
|  | Lombok (7) | 4.42 | -0.08 (-1.19, 1.02) |  |  |  |
| **Sex** | | | | | | |
|  | Female (7) | 4.84 | reference | 0.881 |  |  |
|  | Male (15) | 4.76 | -0.08 (-1.14, 0.98) |  |  |  |
| **Residence** | | | | | | |
|  | Urban (14) | 4.30 | reference | 0.003 | reference | <0.001 |
|  | Semi-rural (8) | 5.63 | 1.32 (0.51, 2.14) |  | 1.86 (1.01, 2.72) |  |
| **Age (months)** | |  | 0.14 (-0.01, 0.30) | 0.065 | -0.02 (-0.15, 0.11) | 0.750 |
| **Stunting^4^** | | | | | | |
|  | No (18) | 4.65 | reference | 0.226 |  |  |
|  | Yes (4) | 5.39 | 0.74 (-0.49, 1.97) |  |  |  |
| **Maternal education** | | | | | | |
|  | Below high school (5) | 4.51 | reference | 0.530 |  |  |
|  | High school and above (17) | 4.87 | -0.36 (-0.81, 1.52) |  |  |  |
| **Parental monthly income^5^** | | | | | | |
|  | ≤ regional minimum salary (11) | 4.49 | reference | 0.202 | reference | 0.584 |
|  | > regional minimum salary (11) | 5.06 | 0.60 (-0.35, 1.54) |  | 0.18 (-0.51, 0.88) |  |
| **Children <5 y in the household** | | | | | | |
|  | 1 (21) | 4.82 | reference | 0.497 |  |  |
|  | 2 or more (1) | 4.04 | -0.78 (-3.11, 1.56) |  |  |  |
| **URTI symptoms^6^** | | | | | | |
|  | No (20) | 4.77 | reference | 0.815 |  |  |
|  | Yes (2) | 4.96 | 0.19 (-1.52, 1.91) |  |  |  |
| **Exposure to cigarette smoke** | | | | | | |
|  | No (13) | 5.04 | reference | 0.186 | reference | 0.179 |
|  | Yes (9) | 4.41 | -.63 (-1.59, 0.33) |  | -0.48 (-1.20, 0.24) |  |
| **Wood-fuelled stove in home** | | |  |  |  |  |
|  | No (20) | 4.86 | reference | 0.353 | reference | 0.023 |
|  | Yes (2) | 4.09 | -0.77 (-2.44, 0.91) |  | -1.68 (-3.10, 0.26) |  |
| ***H. influenzae* carriage** | | |  |  |  |  |
|  | No (20) | 4.75 | reference | 0.599 |  |  |
|  | Yes (2) | 5.18 | 0.44 (-1.27, 2.14) |  |  |  |
| ***S. pneumoniae* carriage** | | |  |  |  |  |
|  | No (14) | 4.73 | reference | 0.756 |  |  |
|  | Yes (8) | 4.88 | 0.15 (-0.87, 1.18) |  |  |  |
| ***M. catarrhalis* carriage** | | |  |  |  |  |
|  | No (21) | 4.72 | reference | 0.165 |  |  |
|  | Yes (1) | 6.27 | 1.56 (-0.70, 3.81) |  |  |  |

^1^*S. aureus* density reported in log_10_ genome equivalents/ml

^2^Coefficient is the difference in means determined by linear regression

^3^Adjusted for residence type, age, income, site cigarette smoke exposure, and wood-fuelled stove

^4^length/height-for-age z-score below -2 standard deviations of the median

^5^Regional minimum salary rates (2016) were 1,800,725 Indonesian rupiah (IDR) in Padang, 2,626,940 IDR in Bandung, and 1,550,000 IDR in Lombok

^6^URTI symptoms include rhinorrhea, cough, and/or tonsilitis

**Table S4. Linear regression analysis of factors associated with the combined density of *S. pneumoniae*, *H. influenzae*, *M. catarrhalis*, and/or *S. aureus* in children colonized with of two or more bacterial species (n=125).**

| **Variable (n)** | | **Mean density (log_10_ GE/ml)^1^** | **Unadjusted coefficient^2^ (95% CI)** | **P value** | **Adjusted coefficient^3^**  **(95% CI)** | **P value** |
| --- | --- | --- | --- | --- | --- | --- |
| **Region** | | | | | | |
|  | Padang (23) | 6.21 | reference | 0.007 | reference | 0.261 |
|  | Bandung (54) | 6.59 | 0.38 (-0.09, 0.85) |  | 0.34 (-0.08, 0.75) |  |
|  | Lombok (48) | 5.98 | -0.22 (-0.70, 0.25) |  | 0.12 (-0.30, 0.52) |  |
| **Sex** | | | | | | |
|  | Female (59) | 6.24 | reference | 0.669 |  |  |
|  | Male (66) | 6.32 | 0.08 (-0.27, 0.42) |  |  |  |
| **Residence** | | | | | | |
|  | Urban (60) | 6.22 | reference | 0.499 | reference | 0.928 |
|  | Semi-rural (65) | 6.34 | 0.12 (-0.23, 0.47) |  | 0.02 (-0.32, 0.35) |  |
| **Age (months)** | |  | -0.03 (-0.08, 0.02) | 0.272 |  |  |
| **Stunting^4^** | | | | | | |
|  | No (80) | 6.15 | reference | 0.040 | reference | 0.079 |
|  | Yes (45) | 6.53 | 0.38 (0.02, 0.73) |  | 0.34 (-0.04, 0.71) |  |
| **Maternal education** | | | | | | |
|  | Below high school (58) | 6.58 | reference | 0.002 | reference | 0.563 |
|  | High school and above (67) | 6.03 | -0.54(-0.88, -0.21) |  | -0.10 (-0.44, 0.24) |  |
| **Parental monthly income^5^** | | | | | | |
|  | ≤ regional minimum salary (83) | 6.39 | reference | 0.073 | reference | 0.481 |
|  | > regional minimum salary (40) | 6.05 | -0.34 (-0.71, 0.03) |  | -0.13 (-0.50, 0.24) |  |
| **Children <5 y in the household** | | | | | | |
|  | 1 (101) | 6.24 | reference | 0.242 |  |  |
|  | 2 or more (24) | 6.50 | 0.26 (-0.18, 0.70) |  |  |  |
| **URTI symptoms^6^** | | | | | | |
|  | No (86) | 6.13 | reference | 0.009 | reference | <0.001 |
|  | Yes (39) | 6.63 | 0.49 (0.12, 0.86) |  | 0.79 (0.42, 1.16) |  |
| **Exposure to cigarette smoke** | | | | | | |
|  | No (75) | 6.26 | reference | 0.746 | reference | 0.268 |
|  | Yes (50) | 6.32 | 0.06 (-0.030, 0.42) |  | 0.18 (-0.14, 0.51) |  |
| **Wood-fuelled stove in home** | | |  |  |  |  |
|  | No (111) | 6.37 | reference | 0.009 | reference | 0.481 |
|  | Yes (14) | 5.64 | -0.73 (-1.27, -0.19) |  | --0.25 (-0.50, 0.24) |  |

^1^*S. aureus* density reported in log_10_ genome equivalents/ml

^2^Coefficient is the difference in means determined by linear regression

^3^Adjusted for region, residence type, stunting, maternal education, income, upper respiratory tract infection (URTI) symptoms, cigarette smoke exposure, and wood-fuelled stove.

^4^length/height-for-age z-score below -2 standard deviations of the median

^5^Regional minimum salary rates (2016) were 1,800,725 Indonesian rupiah (IDR) in Padang, 2,626,940 IDR in Bandung, and 1,550,000 IDR in Lombok

^6^URTI symptoms include rhinorrhea, cough, and/or tonsilitis

**References**

1. Carvalho Mda G, Tondella ML, McCaustland K, Weidlich L, McGee L, Mayer LW, et al. Evaluation and improvement of real-time PCR assays targeting *lytA*, *ply*, and *psaA* genes for detection of pneumococcal DNA. *J Clin Microbiol* 2007, 45(8):2460-2466.
